# Seasonal nutrient contribution of mangrove aquatic foods to fisher households in West Kalimantan, Indonesia

**DOI:** 10.1186/s12889-025-21952-9

**Published:** 2025-05-13

**Authors:** Lucinda Middleton, Puji Astuti, Siti Nurokhmah, Benjamin Michael Brown, Shakuntala Thilsted, Julie Brimblecombe, Natasha Stacey

**Affiliations:** 1 Research Institute for the Environment and Livelihoods, Charles Darwin University, Darwin Northern Territory, Darwin, Australia; 2 Department of Biochemistry and Biomolecular Science, Faculty of Medicine, Universitas Tanjungpura, Pontianak, Indonesia; 3 Department of Nutrition, Faculty of Medicine, Universitas Indonesia, Jakarta, Indonesia; 4 Department of Nutrition Science, Faculty of Health Science, Universitas Muhammadiyah Surakarta, Surakarta, Indonesia; 5 Australian Institute of Marine Science, Darwin, NT Australia; 6 CGIAR, Washington, D. C USA; 7 Department of Nutrition, Dietetics and Food, Faculty of Medicine, Nursing and Health Sciences, Monash University, Melbourne, Australia

**Keywords:** Mangroves, Aquatic foods, Coastal community, Seasonal nutrient intake, Recommended nutrient intake, Indonesia

## Abstract

**Background:**

Aquatic foods are micronutrient-rich and utilised by coastal communities across the globe. However, the contribution of aquatic foods sourced from mangroves to nutrient intake is relatively unknown, despite thousands of people reliant on their resources in coastal regions across the globe. This case study aimed to quantify the nutrient contribution that aquatic foods make to mangrove fishers’ household dietary requirements in a community in West Kalimantan, Indonesia.

**Methods:**

A seven-day household weighed food record of all aquatic food consumed was conducted twice to capture seasonal variability, in the wet (*n* = 59) and dry seasons (*n* = 54). Records were analysed using nutrition composition datasets for finfish and shellfish. The contribution aquatic foods make to the recommended nutrient intake (RNI) was described for seven nutrients: calcium, iron, selenium, zinc, vitamin A, omega-3 essential fatty acids and protein. The total quantity of each species consumed for each season was determined to calculate the average per-person nutrient intake from each species and from all aquatic food species combined. We then compared these to each of the RNI sex and age categories and aggregated it to present an average (%) RNI for the total sample and by season.

**Results:**

Households consumed more meals containing aquatic food in the dry season (390 meals) compared to the wet season (337 meals). Aquatic foods contributed to all seven nutrients analysed, mostly to the RNIs for selenium (127% wet season and 193% dry season), protein (27% wet season and 35% dry season), omega-3 essential fatty acids (21% in both seasons), and zinc (10% wet season and 17% dry season). Contribution to iron reduced from 11 to 10% between the wet and dry seasons and increased from 8 to 10% for calcium and 4–7% for vitamin A between the wet and dry seasons respectively.

**Conclusions:**

Our findings indicate that mangrove aquatic foods provide important nutrients in local seasonal diets in West Kalimantan. Given the nutritional challenges Indonesia faces, sustaining local engagement with mangroves as a food system should be considered in the aquatic foods discourse and nutrition projects, as well as mangrove conservation and management strategies.

**Supplementary Information:**

The online version contains supplementary material available at 10.1186/s12889-025-21952-9.

## Background

Inequities in power and wealth across the globe have contributed to some populations experiencing high burdens of food insecurity and related nutrient deficiencies [1, 2]). In 2023, it was estimated that 2.33 billion people, approximately 29% of the world’s population, experience moderate to severe food insecurity, with 757 million people potentially experiencing hunger [2]. Over 50% of children under five years have at least one micronutrient deficiency in vitamin A, zinc or iron. These are the micronutrients we have evidence of, and it is expected deficiencies are high for other nutrients as well [3]. The consequences of undernutrition can impact the cognitive and physical development of children and their well-being throughout the lifecycle and adversely impact adults as well as societies [4]. Certain gender and age groups, such as pregnant and lactating women and children under 24 and 59 months, have higher risks, especially for micronutrient deficiencies [5]. Furthermore, an increase in the double burden of malnutrition, especially in low- and middle-income countries (LMICs), which is defined by the co-existence of over- and undernutrition, makes addressing of nutrition-related issues more complicated and creates further stressors on health care systems [6].

Indonesia is the largest archipelagic country in the world and is home to 17,000 islands and a diversity of coastal ecosystems and habitats [7]. Indonesia has a complex colonial history [8] and has, in the last two decades, experienced rapid urbanisation and globalisation with dramatic changes in the food environment. These have contributed to both under- and over-nutrition [9]. It has one of the highest stunting rates in the world, impacting 31% of children under five years of age (2022) [10]. Furthermore, 31.2% of women of reproductive age are anaemic, and it is estimated that 4.9% of the Indonesian population experience moderate to severe food insecurity [10]. West Kalimantan, where the study reported herein was conducted, has some of the highest rates of undernutrition across the nation, with 10.3% of children under five years wasted and 21.9% stunted [11]. Further, Kubu Raya Regency in West Kalimantan has one of the highest rates of stunting in the province [12]. The Demographic Health Survey (2017) from Kubu Raya Regency found that 82.9% of children between 6 and 23 months consumed vitamin A-rich foods, and 75.5% consumed iron-rich foods in the preceding 24 h [13]. However, only 49% and 46% consumed four or more food groups, respectively (an indicator of adequate diet diversity), and met minimum meal frequency [13]. To add to this already high burden of undernutrition in Indonesia, there is an increase in the number of adults and children suffering from nutrition-related chronic diseases such as diabetes, obesity, and hypertension [14]. Therefore, finding sustainable and geographically based solutions to both under and overnutrition is essential, especially as Indonesia is experiencing rapid population growth and an increase in detrimental climate events [15].

There has been a push in recent years by research institutes, international agencies, and non-governmental organisations to pay more attention to the potential role aquatic foods can play in combating food and nutrition insecurity, including micronutrient deficiencies, particularly in LMICs [16–18]. Aquatic foods include finfish, shellfish, invertebrates, plants, and any other foods sourced from marine and freshwater environments [19]. Aquatic foods are extremely nutrient-rich, especially in micronutrients such as calcium, zinc, iron, vitamin A and vitamin B12 [20]. Fish, in particular, are rich in fatty acids, which play a vital role in the cognitive development of children [20]. Globally, the small-scale fisheries sector has been found to contribute up to 32% of the aquatic food nutrient supply, providing for millions of people [21]. Recent research has highlighted that nutrients found in small-scale fisheries catch can exceed the recommended dietary intake for communities living within 100 km of the coast and thus help to combat micronutrient deficiencies, especially in vulnerable groups [17, 22].

Coastal communities across Indonesia are highly dependent on marine resources and coastal ecosystems, such as mangroves, for food and nutrition security [23]. As expected in an archipelagic nation, fish and other aquatic foods are the most consumed sources of animal protein in coastal communities across Indonesia [24]. However, the intake of aquatic foods varies greatly across Indonesia, which is influenced by individual and cultural preferences and seasonality [25]. Despite research demonstrating the importance of aquatic foods as an essential dietary source of micro- and macronutrients, especially in LMICs [26], there is still limited research globally on the nutrient value of aquatic foods and their contribution to nutrient intakes, specifically in Indonesia. This risks aquatic foods being overlooked in government and non-government social and environmental policies for their contribution to food and nutrient security for large portions of populations. Ickowitz et al. (2023) found in Indonesia that households consumed 28% more fish if they were located near high-density mangroves compared to communities not located near the mangroves, thus demonstrating that mangroves contribute to local fish consumption and food and nutrition security in Indonesia [27]. In Kubu Raya, West Kalimantan, mangroves are an important local food system and provide over 250 edible species as well as income to the surrounding community [28]. While there is a growing body of evidence on the contribution of aquatic foods to nutrient intake, there is little evidence on the contribution to nutrient intakes of aquatic foods sourced from mangroves. This paper quantifies the nutrient contribution that aquatic foods make to mangrove fishers’ household dietary requirements in a community located in West Kalimantan, Indonesia. This case study answers three research questions; (1) Which finfish and shellfish are gathered, by whom and from what source for home consumption? (2) What nutrient contribution do these aquatic foods from mangroves make to dietary requirements? And (3) Do consumption patterns differ by season, and if so, why? In this study, finfish and shellfish are referred to as aquatic foods. This study contributes to and builds on this evidence to highlight the potential role mangrove systems have in supporting food and nutrition security through income-generating pathways in Indonesia.

## Methods

### Study design

The results reported on in this paper were part of a larger case study project assessing the contribution of mangrove food systems to food and nutrition security. This study used a cross-sectional design conducted with household participants and visitors consuming meals with aquatic foods at two different time points to represent two seasonal periods, the wet and dry seasons. Seven-day weighed food records were used to collect data on household demographics and source and consumption of aquatic foods.

### Field team and approach

As the study was led by a researcher from a Western academic institute, a collaborative approach was taken to move towards decentralising the Western research approach and reduce unconscious bias and inequity [29]. Forming a research partnership with a University in West Kalimantan and building a team of Indonesian researchers were key to this approach. The field team consisted of the lead author, the second author and two enumerators, one for each round of data collection. The lead author is a woman and a British Hong Konger, based at an Australian University, and the second author is a Javanese woman from Pontianak, West Kalimantan, based at the Universitas Tanjungpura (UNTAN). The enumerators were Indonesian and recent graduates from UNTAN and Polytechnic ‘Aisyiyah Pontianak. As a team, we worked collaboratively to include different perspectives and knowledge in the design, implementation, and data analysis of this study. To build trust with the study participants and the local community, several visits were made to field sites before data collection commenced to enable a relationship-building process. We also worked to ensure all research participants were comfortable with the study taking place and that data collection was culturally appropriate and followed any local protocols. The team sought permission from the regional and local government and leaders within each sub-village of Kubu Raya to conduct the study, and all participants gave their free and informed consent.

### Study site

**Fig. 1:**
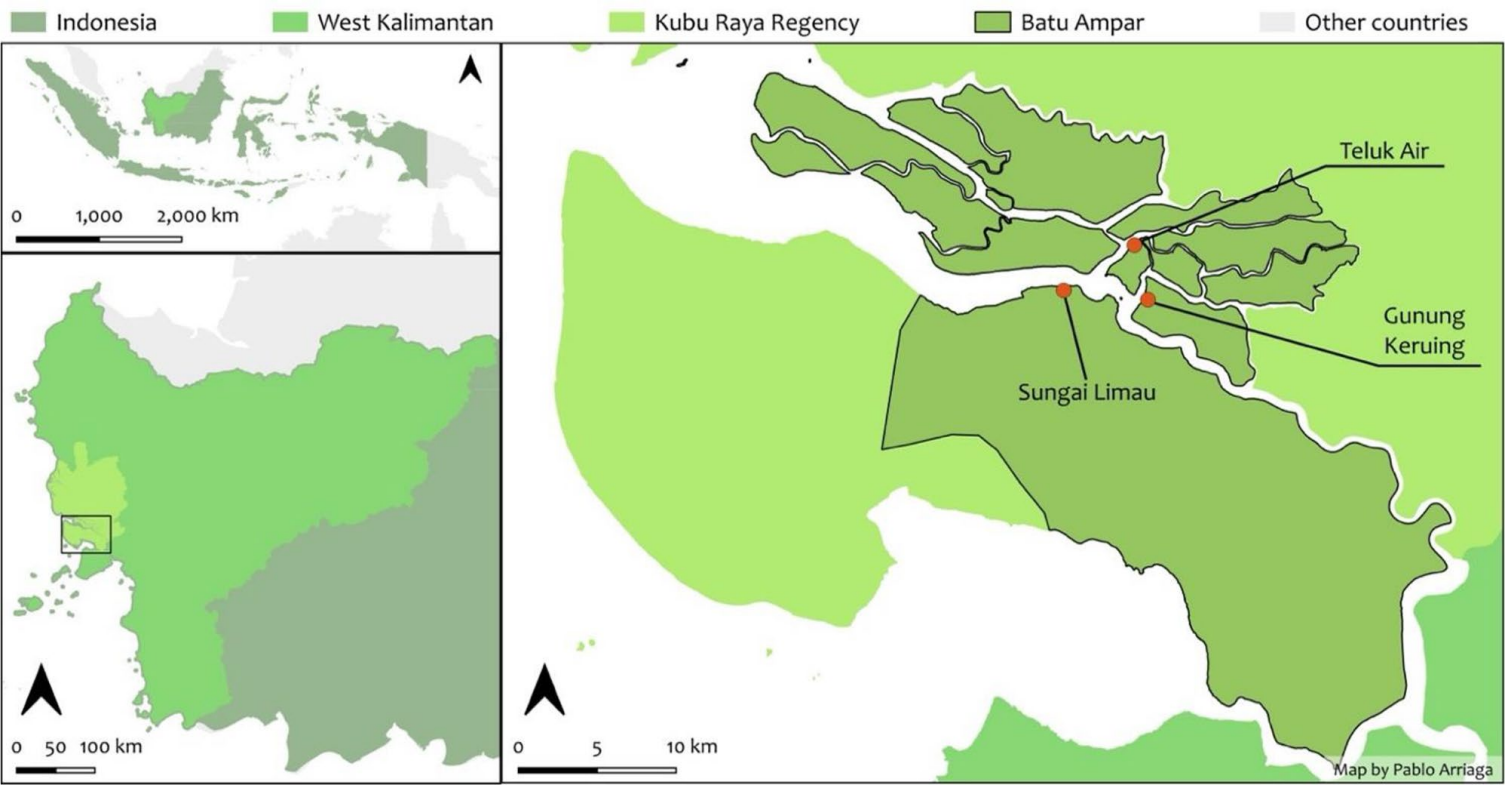
Map of West Kalimantan, Indonesia (top), with sub-village field sites: Teluk Air, Sungai Limau and Gunung Keruing in Batu Ampar Village, Kubu Raya, West Kalimantan

This case study took place in Batu Ampar village (Fig. 1), located in Kubu Raya Regency, West Kalimantan, Indonesia. Batu Ampar is within and adjacent to an extensive mangrove system, which is common to much of the West Kalimantan coastline, which is covered by 161,967 hectares of mangroves [30]. Batu Ampar has 15 sub-villages, with this study focusing on three sub-villages: Sungai Limau, Gunung Keruing and Teluk Air. These three sub-villages were selected for this study due to their proximity to the surrounding mangroves, including the river, mudflats, deltas, and people’s engagement in fisheries activities.

### Sampling

The sample for this study was selected from a larger sample of 115 households as this study is part of a larger project examining the contribution of mangroves to gendered food and nutrition security in West Kalimantan [28]. Purposive snowball sampling was used to select households from Batu Ampar to complete seven-day weighed food records (WFR). The inclusion criteria were households across the three sub-villages of Batu Ampar with (1) members engaged in fishing activities for either subsistence or income generation; (2) children under 18 years of age living at home during the first round of data collection; (3) food consumed from the same source. To commence sampling, a list of 115 households from a larger project related to mangroves and food security for each dusun was randomised in Microsoft Excel, and the first household was visited from each list. We then visited the first households on the randomised list, and each household was asked to participate in the study if they met the above inclusion criteria. This pattern was repeated in each dusun until there were no more households on the list that met the inclusion criteria. The seven-day weighed food record (WFR) was completed twice over an annual period to capture the seasonal variability of finfish and shellfish intake in the wet and dry seasons. The wet season, known locally as the *Barat* (West) season, is from October to January and is marked by an increase in rain, waves, and wind. The dry season, known locally as the *Selatan* (South) season, is between May and September [28]. The first round of data collection took place between 8th and 26th October 2022, during the wet season, and 59 households completed the collection of WFR and demographic information. The second round of data collection took place from 29th May to 8th June 2023, during the dry season, and 54 of the same households completed the food record (Table 1). Four households withdrew from the study due to sickness (2), family disputes (1) and death in the family (1).

### Household seven-day weighed food record

The seven-day WFR is considered the gold standard for dietary assessment [31]. However, it is often designed for an individual and not a household. However, most people in Batu Ampar do not consume food alone but rather consume food communally with household members and visitors or extended family. Further, many households invite other people to share their food. Therefore, an individual seven-day WFR was not appropriate for this setting. Taking this into account and in consultation with the community, we adapted the seven-day WFR to apply to a family household context and sought feedback on a draft WFR and its feasibility. We designed the WFR to be completed by the household food preparer or another household member. All food preparers or nominated household members were over the age of 18.

Information was recorded by the household food preparer or household member on the number of adults and children consuming the meal, their gender and age in addition to the aquatic food species consumed, the quantity consumed in grams, and where the food was sourced (e.g. market or mangroves). Each line of the WRF completed by the food preparer represented a different meal. Data were also collected on the gender of the person or people sourcing the aquatic foods. For example, it was common for women and men to go together to conduct fishing activities or the market to source aquatic foods. Data also included where the aquatic foods were sourced from, such as mangroves or the local market. Each household was given an electric scale (brand and origin, e.g. Excellent Scale Co., Ltd., Jakarta, Indonesia), which was calibrated, checked, and renewed between seasons if necessary. Each electric scale was calibrated when first purchased in September 2022 by weighing the same smartphone twice. They were then checked again once in the study sites using the same smartphone in October 2022, whilst households were being trained to use the WFR. Before the second round of data collection, the team conducted a preliminary visit with households in May 2023 to check the scales using the same smartphone method. If a measurement error occurred, or the scales were broken, new scales were purchased from the same seller and calibrated following the same protocol. Households were also asked to record information on non-regular household members, defined as people not living under the same roof who consumed the meal in the record during the 7 days.

Food was measured as a net weight that would be eaten by the household (including non-regular members). To ensure we reduced measurement errors, we piloted our WFR method over three days. The team trained every household within the sample from each *dusun* on the first day, including in-depth training on how to use the weighing scales. During the following two days, the team revisited each household, re-trained and asked for feedback on their use of the WFR. Each household’s entries were checked to ensure they were confident to complete the record. Households then independently completed the WFR over the next seven days. During the dry season data collection, each household was visited the month before data collection to check their scales and check their availability to participate for a second time. After confirmation of participation and checking of equipment, household members were retrained at the beginning of the seven-day data collection period.

Upon completing the WFR, at the end of the seven days, the team also asked each household a set of endline questions about whether the preceding week mirrored regular consumption patterns. Households could state whether they consumed the same number of aquatic foods or higher or lower amounts than usual. If consumption differed from usual consumption patterns, they were then asked to provide details (open-ended answers) on why their diets had changed.

### Analysis

While the endline questionnaire was being conducted, initial quality control of each WFR was completed by examining the data and identifying outliers, missing data, or likely errors, which were then corrected or confirmed by the food preparer. The resulting data were compiled in Microsoft Excel and analysed in STATA 15. Scientific names were matched to the local names recorded in the WFRs based on a list of aquatic food species sourced from mangroves in Batu Ampar generated by Middleton et al. [28] (Supplementary material dataset).

The nutrient composition of each of the recorded species (based on 100 g of edible portion) was derived from the (1) FAO/INFOODs global composition database for fish and shellfish [32] or (2) FishBase Nutrients, using the Hicks et al. model [17]. If a species was listed in both datasets, the FAO/INFOODs global composition database was used. Based on the key micronutrient deficiencies recognised in Indonesia, especially among children under five years [33], seven nutrients were included in this analysis: calcium (Ca), iron (Fe), selenium (Se), zinc (Zn), vitamin A (Vit A), omega-3 essential fatty acids and protein. Nutrient values selected accounted for the edible portion, the form of fish (fresh, dry, preserved), and the cooking method (dry, moist).

Data were analysed descriptively. First, the total (wet weight equivalent) quantity of each species consumed for each of the seven days combined, and for all households combined, by gender, by source and by season was determined. Wet weight conversion factors were derived from the Indonesian Ministry of Health [34] and the FAO/INFOODs for each species (Supplementary Information Table S3). The total (wet weight equivalent) quantities for each species were then summed to derive a total aquatic (wet weight equivalent) intake for the total sample, by gender and by season. Wet weight equivalent per person per day (by source, gender and season) was derived by dividing the total aquatic (wet weight equivalent) intake by the total sample (household and non-household members for all households combined). The total nutrient intake by species was calculated for each of the seven days based on multiplying the total quantity of each species consumed by the species nutrient value per 100-gram edible portion. This was derived for both seasons. We also adjusted the quantity using a yield factor which was the ratio of the weight of the aquatic food after preparation (cooked, processed) to its weight before preparation, as some composition data were only available in the raw form (Supplementary information Table S2). The total nutrient intake for each species consumed was then divided by the total sample to give an average total per person nutrient intake for each of the species for each of the seven days. The average per-person nutrient intake for each species were then aggregated to give the total aquatic nutrient intake per person per day for each of the seven days. The average total per person nutrient intake for each species and total aquatic nutrient intake per person per day were then compared to each of the RNI sex and age categories relevant to the sample (Indonesian tables, Supplementary Information Table S1) for each of the study nutrients. Each member of the total sample for each season was matched to an RNI sex and age category. These percentages for each species and total aquatic nutrient intake were then summed and divided by seven to give an average per day percentage contribution of species and total aquatic nutrient intake to RNI. These calculations were done for each season.

The quantities of aquatic foods used in the calculation were in grams adjusted by the form (fresh, dried) and cooking methods (dried, moist) of the fish consumed using a conversion factor (weight yield factor) (Supplementary Information Table 2) [13]. If the weight yield factor and wet weight equivalent were missing, we used the value from a substitute species selected based on the size and genus (Supplementary Information Table 3). For large fish, we used the nutrient makeup of fillets, even though some family members may have eaten the head or tail. Endline questions regarding relative consumption patterns were collected from households after the seven-day food record period. The open-ended questions were inputted into Microsoft Excel and analysed by conducting inductive coding. Open-ended answers were inductively placed into categories and finalised.

## Results

The first round of data collection took place between the 8th and 26th of October 2022, during the wet season, and 59 households completed the collection of WFR and demographic information. The second round of data collection took place from 29th May to 8th June 2023, during the dry season, and 54 of the same households completed the food record (Table 1). Four households withdrew from the study due to sickness (2), family disputes (1) and death in the family (1). Five households did not consume aquatic foods in both seasons. The average household size was 5.5 members and 5.4 members in the wet and dry seasons, respectively. Household members were between 6 months old and 50 and above. Most household members were above the age of 18 (Table 1).

**Table 1:** Summary of the demographic characteristics of households in Batu Ampar

Characteristics	Wet season	Dry season
Number of households	59	54*
Number of households by *dusun*		
*Gunung Keruing*	20	18
*Teluk Air*	19	19
*Sungai Limau*	20	17
Household size, mean ± SD	5.5 ± 1.8	5.4 ± 1.8
Total number of household members*	*N* = 306	*N* = 282
Age (years) of family members, n (%)		
0–4	34 (11.3)	32 (11.6)
5–9	48 (15.9)	44 (15.9)
10–17	57 (18.9)	51 (18.4)
18–49	138 (45.8)	126 (45.5)
50 or above	29 (9.6)	24 (8.7)
Gender of household members		
Female	145 (48.2)	135 (48.7)
Male	161 (53.5)	147 (53.1)

*Data were collected using household measurements but are presented as per person and include both household members and non-formal members (visitors) *lost to follow-up (*n* = 5 households)

**Fig. 2:**
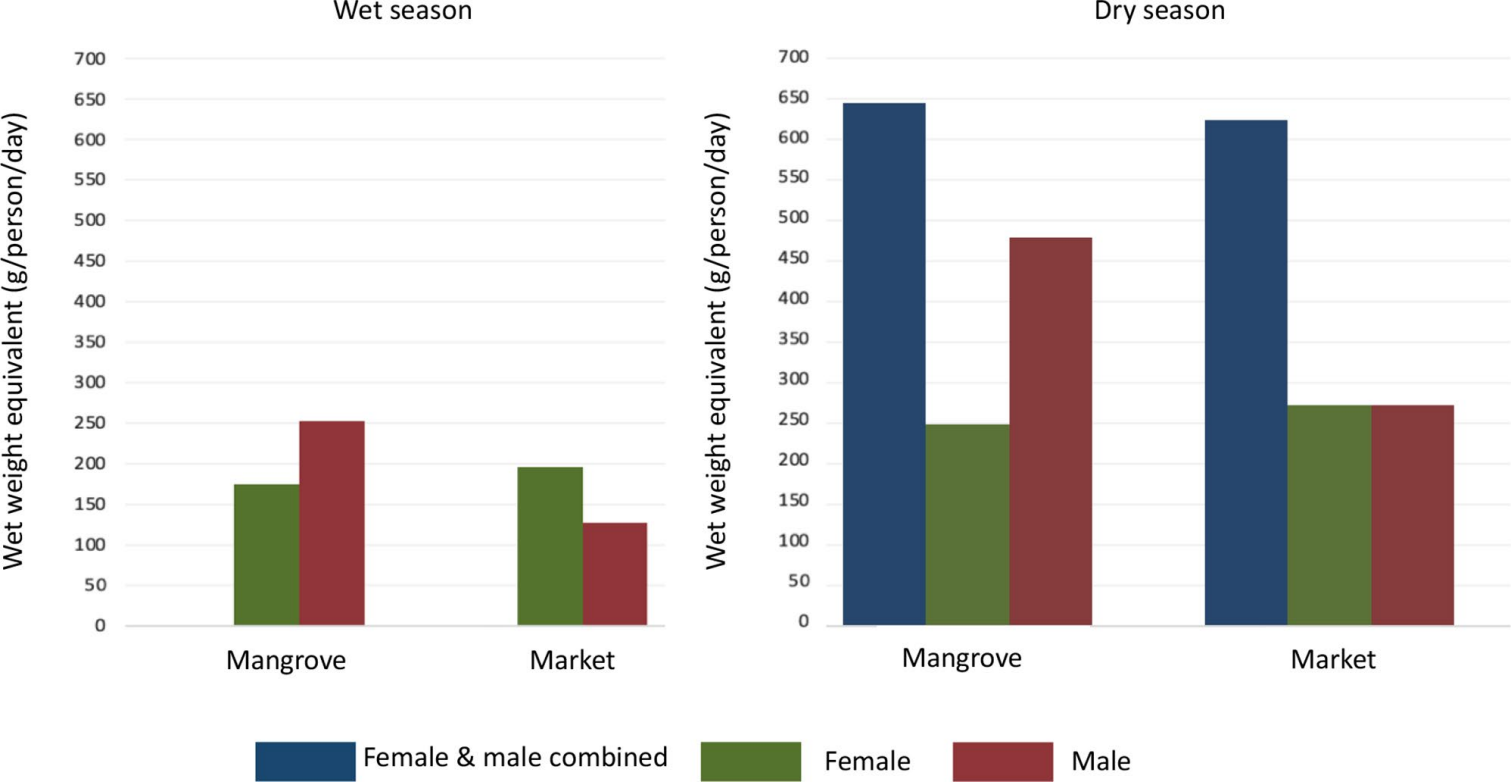
Seasonal average wet weight equivalent (g/person/day) of aquatic foods consumed according to source (market/mangrove) and gender (Female/Male or Female & Male) of the person responsible for sourcing the aquatic foods. **(a)** represents the wet season **(b)** represents the dry season

A greater diversity of aquatic food species were consumed by the total sample in the wet season (50 species) compared to the dry season (33 species); however, the number of times aquatic foods were consumed was greater in the dry season (Fig. 2). Households consumed 390 meals containing aquatic foods over the 7 days in the dry season, which is 53 more meals than in the wet season. The average consumption of aquatic foods (wet weight equivalent to grams per capita per day) was higher in the dry season compared to the wet season, with the majority of aquatic foods sourced from the surrounding mangroves compared to the market (Fig. 2).

Women and men worked together to source aquatic foods in the dry season, whereas, in the wet season, our data show that women and men sourced aquatic foods separately (Fig. 2). Male household members sourced more aquatic foods from mangroves in both seasons, compared to women, and sourced less food from the market for their households in the wet season and the same as women in the dry season (Fig. 2).

**Fig. 3:**
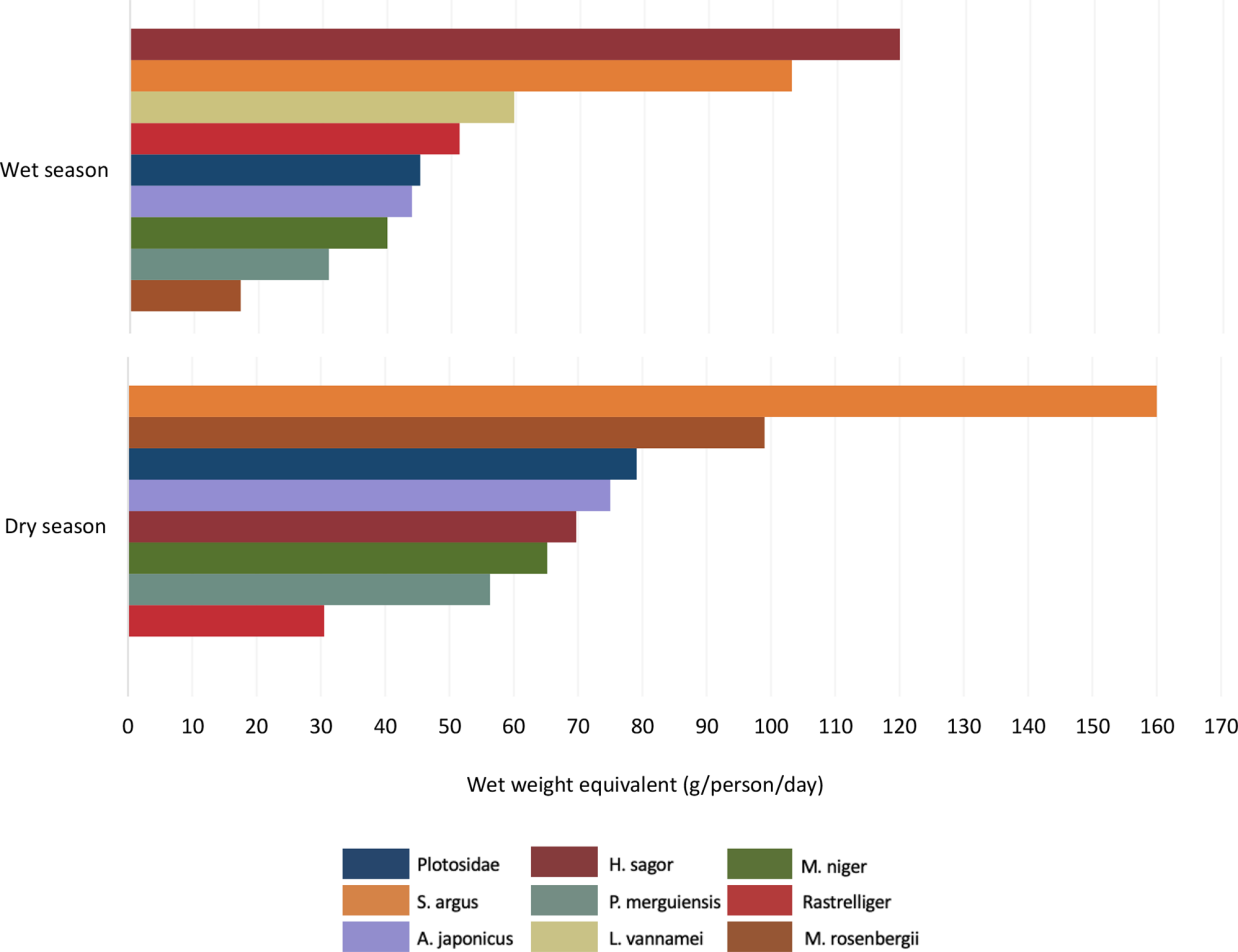
Seasonal trends in the wet weight equivalent (grams) consumption of the top nine aquatic food species consumed per person per day

Participants most frequently consumed nine and eight species of aquatic foods in the wet and dry seasons, respectively (Fig. 3). Of the top nine species consumed, three were shellfish: M.rosenbergii (*Macrobrachium. Rosenbergii*/giant freshwater shrimp), P.merguiensis *(Penaeus merguiensis*/shrimp), and L.vannamei (*Litopenaeus vannamei/*whiteleg shrimp), and six were finfish; Plotosidae (eeltail catfish), by S.agrus (*Scatophagus argus)*, A.japonicus (*Argyrosomus japonicus/*jewfish), H.sagor (*Hexanematichthys sagor*/marine catfish), M.niger (*Macolor niger/*black and white snapper), and Rastrelliger (chub mackerels), in both the wet and dry season. Approximately 120 g per capita per day of *Hexanematichthys sagor* (marine catfish) was consumed in the wet season, making it the most consumed species of the observation period, followed by *Scatophagus argus* (spotted scar/butterfish); which was the most consumed species in the dry season observation period (130 g/person/day). In the dry season, there was no consumption of *Litopenaeus vannamei* (whiteleg shrimp) (Fig. 3).

**Fig. 4:**
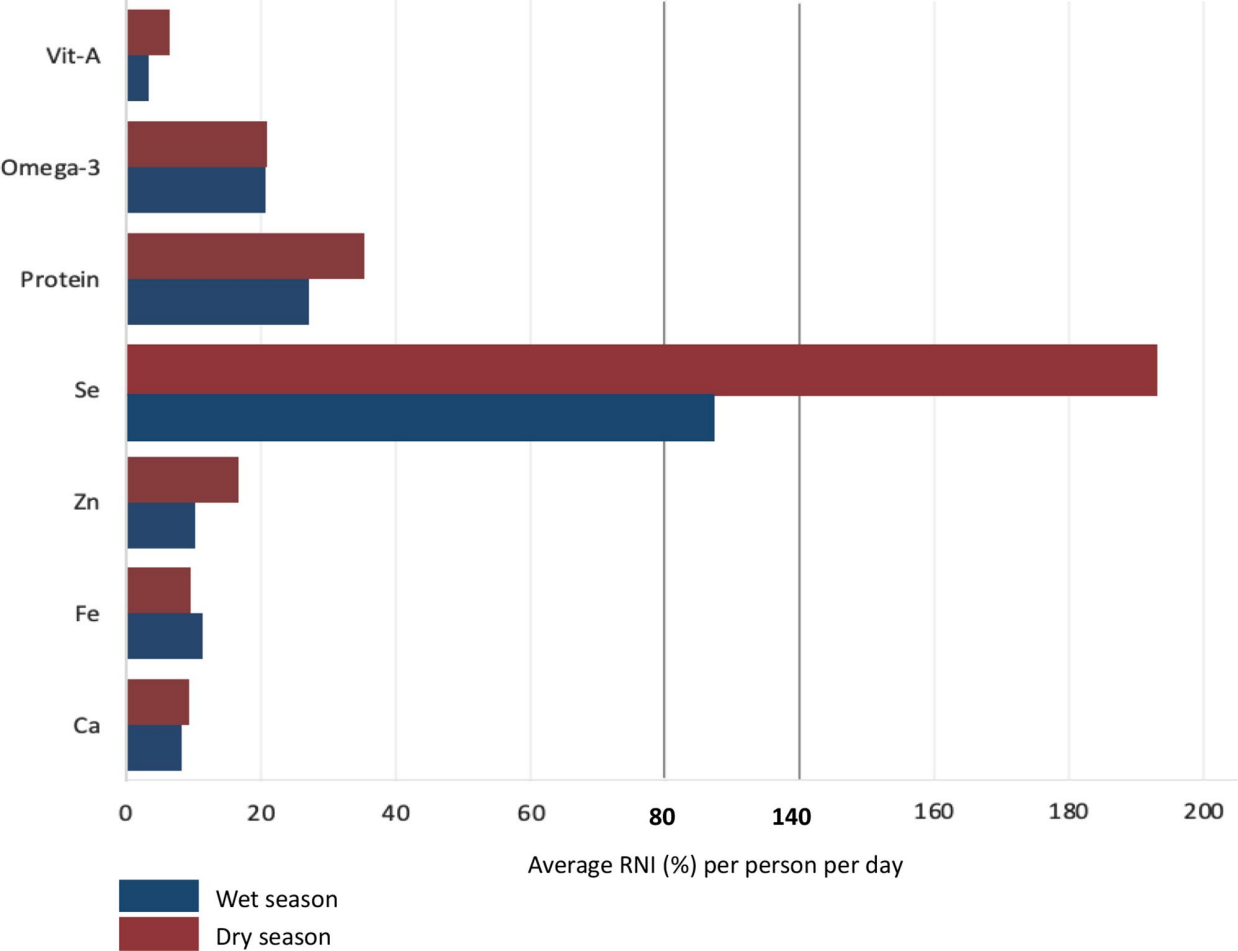
Contribution of aquatic foods (all species consumed for each season) in meeting dietary requirements as a percentage of recommended nutrient intake (RNI), for each nutrient on average per person per day, by season

Selenium intake from aquatic foods was very high in both seasons, with the results suggesting that, on average, the average per capita per day consumption was over 100% of the per person average RNI for selenium (Fig. 4). The percentage of the per person average RNI for vitamin A from aquatic foods was the lowest compared to the other micro- and macronutrients at an average of 4% and 7% in the wet and dry seasons, respectively. The intake of omega-3 essential fatty acids from aquatic foods did not differ greatly between seasons, being relatively stable at an average of around 21% of the per-person average RNI. Omega-3 essential fatty acids were the only nutrient with the percentage contribution to the RNI being the same in both seasons; for all other nutrients, the intakes from aquatic foods increased in the dry season. Calcium intake from aquatic foods was higher in the dry season compared to the wet (10% vs. 8%) (Fig. 4). Zinc intake from aquatic foods, like protein, was higher in the dry season compared to the wet season (17% vs. 10%). Iron was the only micronutrient where the intake from aquatic foods was lower in the dry season, however, the reduction in the contribution to RNI was marginal at 1% point less (11% vs. 10%).

**Fig. 5:**
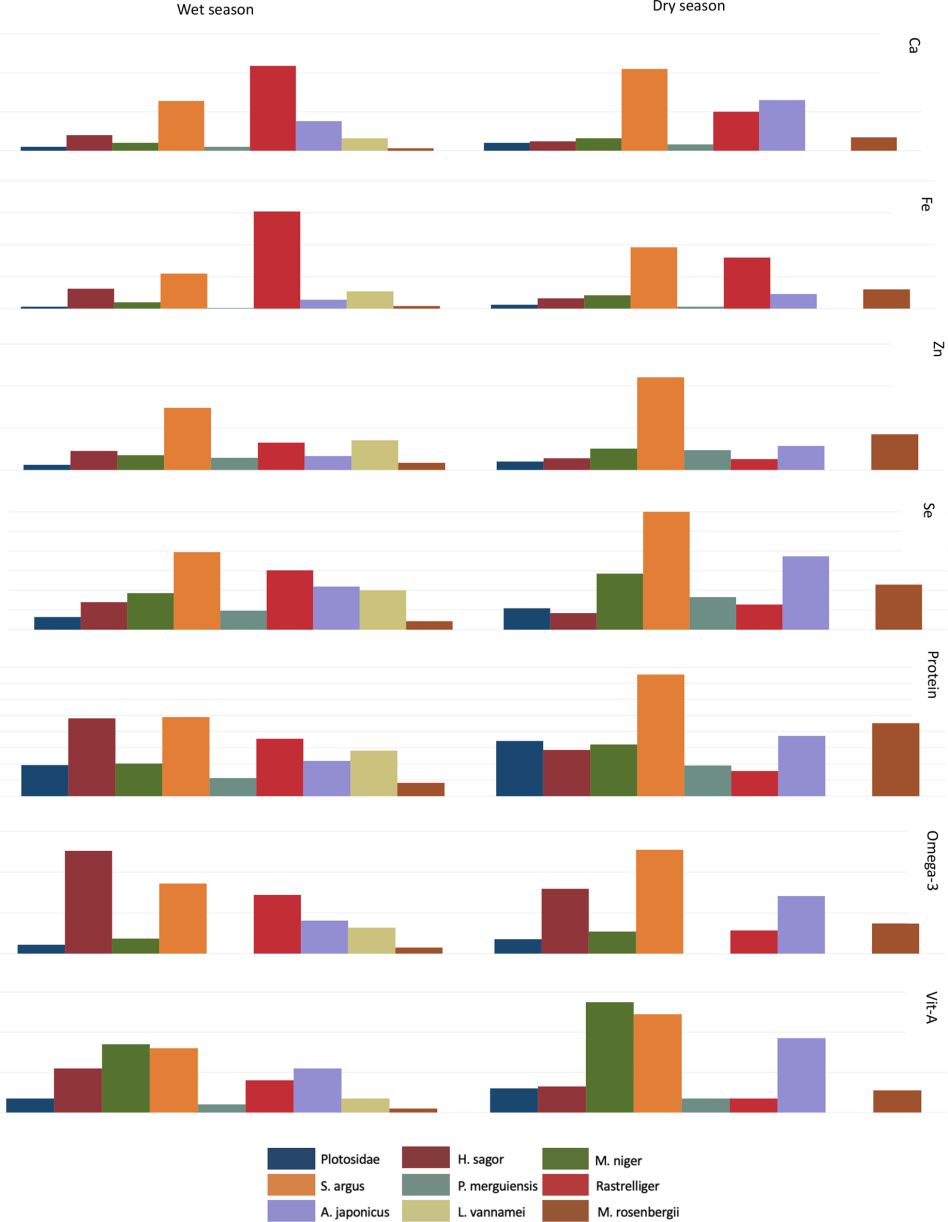
Seasonal contribution of the top nine aquatic food species consumed in meeting dietary requirements as a percentage (%) of the per person average recommended nutrient intake (RNI) for 7 nutrients per person per day

As expected, in line with an increase in spotted scar/butterfish consumption in the dry season, this species contributed the greatest to the per-person average RNI of all nutrients, except vitamin A in the dry season (Fig. 5). Spotted scar/butterfish also contributed to the per person average RNI in the wet season, as did marine catfish, especially to macronutrient (omega-3 essential fatty acids and protein) intakes. Chub mackerels contributed to the per-person average RNI of iron and calcium, especially in the wet season. The contribution of all species to the percentage per person average RNI for vitamin A was lower than that for other nutrients. No species contributed more than 6%, with black and white snapper contributing the greatest to the per person average RNI compared to other species, especially in the dry season.

**Fig. 6:**
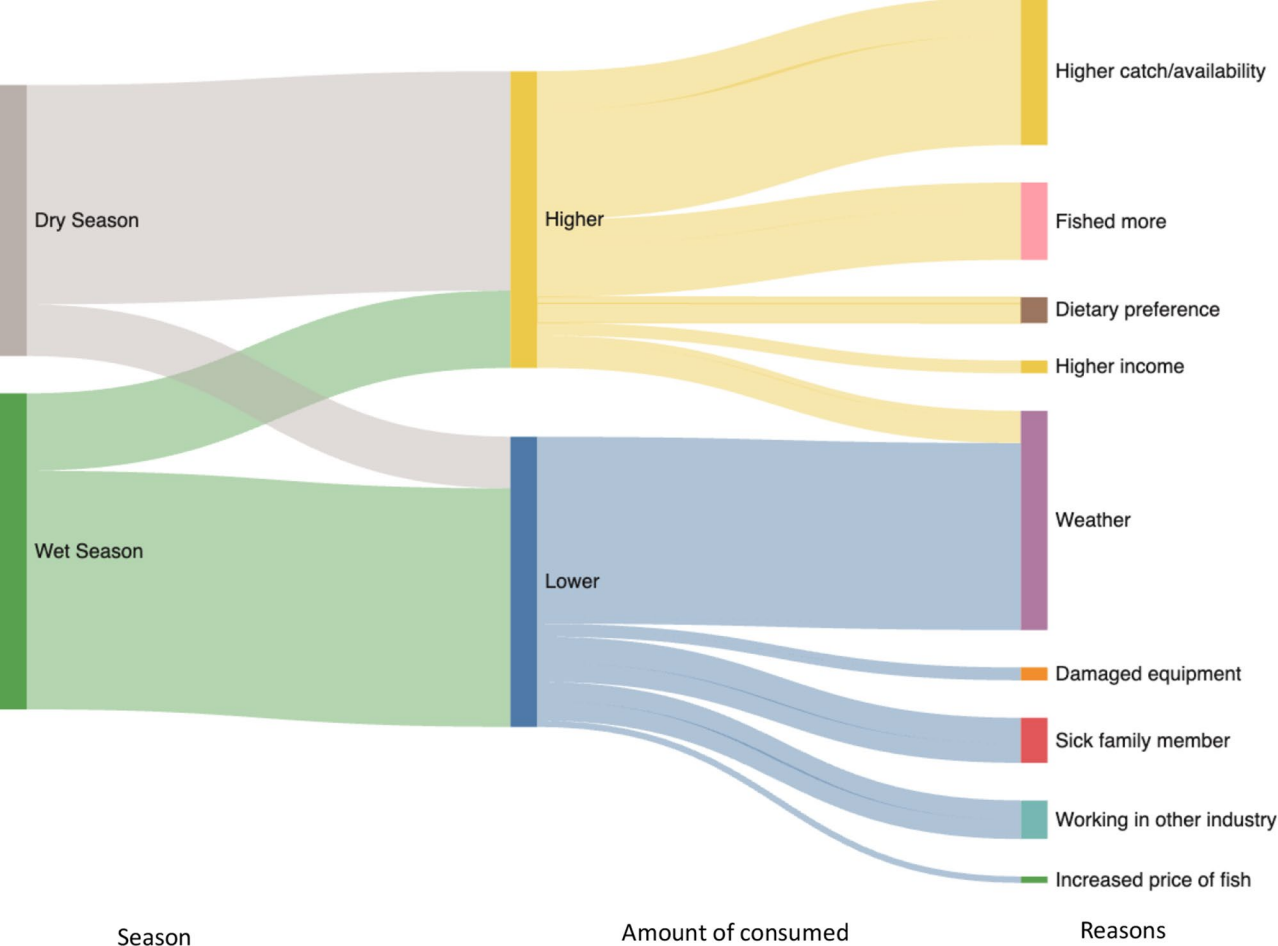
Seasonal trends in the amount of aquatic foods households consume with reasons

The reasons for changes in consumption of aquatic foods varied and included: weather including poor conditions for fishing, income changes such as earning more, and increased availability of aquatic foods in the dry season (Fig. 6). In the wet season, households stated the weather, such as rain, lightning and wind affecting harvesting had the largest impact on reducing the dietary intake of aquatic foods, along with working in other livelihood activities, having a sick family member, increased price of fish and issues with fishing equipment (broken and damaged). In contrast, higher availability, or a greater quantity of aquatic foods caught in the dry season combined with spending more time fishing were cited as the main reasons for consuming more aquatic foods, in the dry season (Fig. 6). For those consuming more than usual in the wet season, higher income earnings and dietary preferences were cited as the main reasons. Income (including working more or in other industries), availability and weather all appeared to be influencers of aquatic food intake (Fig. 6).

## Discussion

Aquatic foods have often been ignored for their current contribution to and potential to support public health and nutrition solutions, despite being consumed by billions of people across the globe and comprising an important part of people’s food systems and knowledge systems that have existed for millennia [35, 36]. Further, the role of mangroves as food systems has largely been left out of the aquatic foods discourse. Our findings suggest that aquatic foods sourced from mangroves support the intake of essential micro- and macronutrients and contribute to the FNS of people in Batu Ampar, West Kalimantan, Indonesia. The findings from this study suggest that a large number and variety of aquatic food species make an important contribution to the diets of people living in Batu Ampar as they were consumed 337 times by households in the wet season and 390 times in the dry season by households (Fig. 2). Ickowitz et al. (2023), using national data from 6741 villages across Indonesia, found that mangrove-dependent communities consumed up to 28% more aquatic foods than communities not residing near mangroves [27]. Previous studies have highlighted how mangroves contribute to aquatic food intake and FNS are important given the recent attention on aquatic foods for their potential to support FNS and reduce malnutrition [16, 37]. This is important as Asia accounted for 72% of the 158 million tonnes of aquatic foods that were available for home consumption globally (2019) [38], which offers countries in Asia, including Indonesia, a unique opportunity to harness the potential of customary procured micronutrient-rich aquatic foods to combat and reduce malnutrition [39]. Furthermore, as Indonesia is home to the greatest density of mangroves globally [40], there could be an opportunity to manage these ecosystems holistically in ways that incorporate FNS benefits. Although not specific to mangroves, there are examples of policy coherence across sectors in Indonesia, as illustrated by the Ministry of Marine Affairs ‘Love Eating Fish’ campaign. The collaborative campaign aims to increase fish consumption to improve nutrition [41] which was announced on National Fish Day in Indonesia (21st November Decree No. 3 2014) which also focuses on raising public awareness of the importance of fish for nutritional intake [42]. This is perhaps an example of the benefits of multisectoral collaboration and that FNS can be integrated into marine policies. Therefore, there could be potential for Indonesia to integrate FNS into existing and future mangrove policies, focusing on improved outcomes for the environment and people.

Much like other global food systems, especially in low to middle-income countries and rural coastal communities, seasonal fluctuations in key nutrient intake were noted in this study. We found that although a greater diversity of aquatic foods were consumed in the wet season, they were consumed in lower quantities. Whereas fewer species were consumed in the dry season but at a much greater quantity per day. Therefore, our findings suggest that seasonality, much like in another context, influences nutrient intake from aquatic foods [43]. We have also shown in Batu Ampar, that community members who utilised mangroves as a food system, listed over 250 species which were also more available in the dry season [28]. Participants from that study also noted weather as a major barrier to mangrove food system utilisation [28]. The findings from this study also add to the evidence, that as consumption of aquatic foods in terms of quantity increased and as expected so did nutrient intake from aquatic foods (Figs. 3 and 4). Further, households listed weather as a major reason for reducing the intake of aquatic foods in the wet season and higher availability and fishing as a reason for increasing intake in the dry season.

Understanding how species sourced from mangroves contribute to nutrient intake is important for the use of aquatic foods in nutrition interventions to reduce micronutrient intake. The body of evidence on aquatic foods and food and nutrition security is growing [16, 17, 39] however, case studies using seasonal seven-day weighed food records and species-specific intake and how these contribute to RNI are not as common. The contribution to the per-person average RNI of selenium was high, in both seasons and increased in the dry season. However, most species contributed between 30% and 100% on average to the selenium RNI, and *Scatophagus argus* contributed over 100% in both seasons. Daily selenium needs are between 20 and 70 µg but could change depending on gender, pregnancy and lactation [44]. Selenium is needed in very small quantities, with recommendations stating that the maximum intake should be limited to 5 mg per person per day [44, 45]. However, a study in Zambia examining the contribution of aquaculture to nutrient intake also noted that participants on average received almost double their RNI for selenium [46]. Of the 37 species of aquatic foods that Hallström et al. (2019) analysed, they found that they contained high amounts of selenium and other micronutrients, and therefore, it was expected that participants may receive more than the RNI [46, 47].

Other nutrients of interest, specifically for Indonesia are iron and the macronutrient, protein. Protein has been highlighted as important during the complementary feeding period (6–23 months), as this period is considered a window of opportunity to prevent stunting [48]. Curbing the stunting rates in Indonesia is a widespread topic across the nation and is considered a top priority for the government, resulting in the stepping up of efforts, in 2017, by creating the National Strategy to Accelerate Stunting Prevention 2018–2024 [49], specifically aiming to improve complementing feeding and reduce micronutrient deficiencies [50]. Therefore, increasing protein intake is vital to prevent stunting, and finding sustainable ways to increase intake is vital in Indonesia [48]. Our findings suggest that aquatic foods are an important source of animal protein and contribute to RNI, with most species contributing between 10% and 40% of the per-person average RNI, in both seasons. Similar to protein intake, iron deficiency is considered to be a major public health concern in Indonesia and several programs mainly aimed at female adolescents, pregnant and lactating women and young children were included in the National Strategy to Accelerate Stunting Preventions 2018–2024 [51]. Although the intake of iron from aquatic foods in this study was not as high as other nutrients, aquatic foods still contributed to the iron RNI. Chub mackerels contributed the most to RNI for iron and were consumed in both seasons. Further, this study measured the intake of community members, from 6 months to 50 years and above. Meaning if iron is essential for different age groups, community members could be consuming iron during important windows of time. Roos et al. (2007) found that local fish in Cambodia could contribute 45% of iron daily requirements for women in rural households [52]. However, an analysis of fish and iron intake in Malawi showed that larger fish had a higher association with anaemia in children under 15 months compared to small fish and that overall fish consumption had only marginal positive impacts on iron status [53]. Most fish consumed by the participants in our study were larger, which may have resulted in lower intakes of micronutrients such as iron and vitamin A which are known to be high in small indigenous fish species. Regardless, there is not one food source that should be relied upon to provide nutrients. Rather a diverse diet, which in this population includes a variety of aquatic mangrove species, is needed to meet nutrient requirements.

Globally vitamin A deficiency (VAD) is a significant public health concern, with governments from countries across low to middle-income countries committing to interventions to reduce the prevalence, through widespread supplementation [54, 55]. Given the devastating impact VAD can have on individuals, solutions that include supplementation, fortification and use of wild, natural foods to increase consumption of beta-carotene and retinol have been suggested [56]. Considering this and that aquatic foods are micronutrient-rich, some have argued that increasing the intake of vitamin A-rich aquatic foods can be part of the long-term solution to reducing VAD. However, findings from our study suggest that the aquatic foods consumed in Batu Ampar were not contributing as much to RNI of vitamin A as they did to other nutrients. Several studies from Bangladesh have shown that small indigenous fish species are very rich in vitamin A and could contribute to RNI [57]. Roos et al. (2003) found that one species, mola, contributed on average 21% of household RNI for vitamin A, throughout the study period [57]. It could therefore be that the most consumed species by households in this study are not as vitamin A-rich as other species, especially as most are medium and large fish species, and the bones and some other parts are not consumed. If there are specific species rich in vitamin A, in Batu Ampar and other regions of West Kalimantan, promoting their consumption could improve vitamin A status. However, if this is to occur, there are harvesting and climatic issues to consider. Vitamin A is also mostly stored in the liver, which we did not account for in the analysis and therefore, the contribution towards vitamin A intake may be underrepresented as may also be the case for iron.

This study provides an initial case study on how aquatic foods sourced from mangroves contribute to the RNI in a small community in Kubu Raya, Indonesia. There is great potential to build on this research and develop a more comprehensive understanding of how mangroves support nutrient intake in different contexts and with different demographic groups. Further research in this area will generate knowledge on mangroves to ensure that the FNS benefits derived from them are not overlooked. This is important given the most recent 2024 State of the World’s Mangroves report acknowledged the FNS benefits derived from mangroves by communities across the globe, an aspect that was not covered in the previous 2022 report [40, 58] This acknowledgement along with various other studies examining fish consumption [27], ecosystem services [59, 60] and FNS benefits [28] from mangroves are an example that these benefits and the associated values should be considered within management and conservation strategies more explicitly. Further, community members should also be included in the policy-making process and the value of local knowledge and governance when considering integrated policies. Therefore, there is a need for further research examining how FNS benefits derived from mangroves can be integrated into management and conservation policies as this has not been explored in depth Further research in this area is also timely given Indonesia’s commitment to mangrove planting and blue carbon [61] and an increasing global interest in blue carbon financing [62]. Increasing our understanding of the FNS value from mangroves is important as they need to be carefully considered within the cost-benefit analysis for communities deciding whether to engage with the credit system, so they don’t lose access to a vital food system.

### Limitations

This was a case study, in one location in Indonesia within a rural community. While it is somewhat representative of coastal communities engaging in mangrove livelihoods in this part of Indonesia that rely on marine resources for food and income, it cannot be considered as being representative of all mangrove-dependent communities. Further, sampling bias may have occurred which can affect the results. We used purposive snowball sampling to identify households that met the inclusion criteria, which are based on referrals and can lead to biased samples as participants tend to include individuals or households with similar characteristics, which can also occur when conducting a case study. It is also acknowledged that children and adults may consume different portion sizes of aquatic foods, given the method of data collection and burdensome to the participants, we did not collect individual portion sizes for each household member and therefore did not account for this difference. This study is reliant on open-sourced nutrient composition data to quantify the nutrient intake of aquatic foods in Batu Ampar. Both uFiSh and FishBase Nutrients are open-sourced datasets and do not contain nutrient information on all species consumed by communities in West Kalimantan. uFiSh is based on laboratory analysis and contains specific data on how the shellfish was cooked and prepared, however, there are a limited number of species included in the dataset. Further, the finfish and shellfish that were analysed in the formation of this dataset are not specifically from Indonesia. FishBase Nutrients includes over 1500 finfish species found specifically in Indonesia; however, values are predictions from a Bayesian hierarchical model, created by Hicks et al. (2019) that includes phylogenetic and trait-based data to predict the concentrations of specific nutrients [17]. This means that the nutrient contents of species are not derived from laboratory analyses of samples and therefore may not be accurate. We also did not collect data on the consumption of other food groups to compare whether aquatic foods were the main animal protein in their diets. There is also a need for more open-access nutrition composition data on aquatic foods from various contexts. This is specifically important for countries wanting to use aquatic foods as part of their approach to tackling micronutrient deficiencies.

## Conclusion

Given that Indonesia has the most extensive area of mangroves globally and faces significant public health and nutritional challenges, the contribution of aquatic foods sourced from mangroves through customary ways should be considered as part of the solution. Integrating aquatic foods into nutrition projects and marine conservation and management strategies, including mangroves, has already been suggested to be a sustainable solution to micronutrient deficiencies [16, 36, 37]. There is a clear emergence in research highlighting the potential of aquatic foods to nourish populations around the globe, specifically those residing near freshwater or marine environments. Our findings suggest that aquatic foods are a staple of the diet and important for supporting micro- and macronutrient intakes. Yet, the current formation of policy and active management of mangroves does not include FNS and there is a need to take perception and attitudes towards mangroves beyond a pure conservation narrative to include the vast benefits communities derive from them as a food system [27, 28, 63]. We, therefore, encourage collaboration between local communities, the scientific community, the private sector and organisations in the development and implementation of mangrove-based strategies that integrate the food values and nutrition benefits communities derive from them.

## Electronic supplementary material

Below is the link to the electronic supplementary material.

Supplementary Material 1

## Data Availability

All data generated or analysed during this study are included in this published article.

## Abbreviations

FAO: Food and Agriculture Organization
FNS: Food and nutrition security
LMICs: Low to middle-income countries
RNI: Recommended nutrient intake
WFR: Weighed food record
VAD: Vitamin A deficiency
