# Supplementary material for: Seasonal nutrient contribution of mangrove aquatic foods to fisher households in West Kalimantan, Indonesia
Source: BMC Public Health. 2025 May 13;25:1764. doi: 10.1186/s12889-025-21952-9 (PMC12070671; doi:10.1186/s12889-025-21952-9)
Supplement: Supplementary file 1 — Supplementary Material 1 [file 12889_2025_21952_MOESM1_ESM.docx]

**Supplementary Information Table S1** Recommended Nutrient Intake (RNI) for seven nutrients by age and gender

| **Gender,**  **Age (years)** | **Protein (g)** | **Calcium (mg)** | **Fe (mg)** | **Se (mcg)** | **Zn (mg)** | **Vit-A (RE)** | **Omega-3 fatty acids (g)** |
| --- | --- | --- | --- | --- | --- | --- | --- |
| *Female* |  |  |  |  |  |  |  |
| 1 - 3 | 20 | 650 | 7 | 18 | 3 | 400 | 0.7 |
| 4 - 6 | 25 | 1000 | 10 | 21 | 5 | 450 | 0.9 |
| 7 - 9 | 40 | 1000 | 10 | 22 | 5 | 500 | 0.9 |
| 10 - 12 | 55 | 1200 | 8 | 19 | 8 | 600 | 1.0 |
| 13 - 15 | 65 | 1200 | 15 | 24 | 9 | 600 | 1.1 |
| 16 - 18 | 65 | 1200 | 15 | 26 | 9 | 600 | 1.1 |
| 19 - 29 | 60 | 1000 | 18 | 24 | 8 | 600 | 1.1 |
| 30 - 49 | 60 | 1000 | 18 | 25 | 8 | 600 | 1.1 |
| 50 - 64 | 60 | 1200 | 18 | 25 | 8 | 600 | 1.1 |
| *Male* |  |  |  |  |  |  |  |
| 1 - 3 | 20 | 650 | 7 | 18 | 3 | 400 | 0.7 |
| 4 - 6 | 25 | 1000 | 10 | 21 | 5 | 450 | 0.9 |
| 7 - 9 | 40 | 1000 | 10 | 22 | 5 | 500 | 0.9 |
| 10 - 12 | 50 | 1200 | 8 | 22 | 8 | 600 | 1.2 |
| 13 - 15 | 70 | 1200 | 11 | 30 | 11 | 600 | 1.6 |
| 16 - 18 | 75 | 1200 | 11 | 36 | 11 | 700 | 1.6 |
| 19 - 29 | 65 | 1000 | 9 | 30 | 11 | 650 | 1.6 |
| 30 - 49 | 65 | 1000 | 9 | 30 | 11 | 650 | 1.6 |
| 50 - 64 | 65 | 1200 | 9 | 30 | 11 | 650 | 1.6 |

RE, retinol equivalent.

**Supplementary Information Table S2** Nutrient composition of fish species by cooking method

| **Species** | **Cooking method** | **Substitute species for yield factor**^2^ | **Yield factor**^2^ | **Form** | **Database** | **Substitute species for nutrient value** | **Ca (mg)** | **Fe (mg)** | **Omega-3 (g)** | **Protein (g)** | **Se (mcg)** | **Vit-A (RE)** | **Zn (mg)** |
| --- | --- | --- | --- | --- | --- | --- | --- | --- | --- | --- | --- | --- | --- |
| Acetes | dry | Not applicable | 1.00 | grilled | ufish | A indicus | 437.000 | 2.700 | 0.000 | 21.500 | 56.977 | 56.512 | 1.291 |
| A chacunda | dry | O niloticus | 0.79 | raw | ina | None | 357.000 | 2.500 | 0.889 | 18.200 | 51.900 | 10.100 | 2.170 |
| A chacunda | moist | O niloticus | 0.87 | raw | ina | None | 357.000 | 2.500 | 0.889 | 18.200 | 51.900 | 10.100 | 2.170 |
| A japonicus | moist | Siluriformes | 0.83 | raw | ina | A amoyensis | 110.000 | 0.525 | 0.289 | 19.200 | 49.300 | 38.500 | 0.841 |
| A japonicus | dry | S salar | 0.86 | raw | ina | A amoyensis | 110.000 | 0.525 | 0.289 | 19.200 | 49.300 | 38.500 | 0.841 |
| C lucius | dry | T trachurus | 0.80 | raw | ina | None | 51.100 | 0.505 | 0.179 | 17.900 | 44.100 | 42.200 | 0.863 |
| C micropeltes | dry | T trachurus | 0.80 | raw | ina | None | 51.100 | 0.505 | 0.179 | 17.900 | 44.100 | 42.200 | 0.863 |
| C chanos | dry | T trachurus | 0.80 | raw | ina | None | 61.900 | 0.672 | 0.248 | 19.400 | 91.100 | 11.300 | 1.340 |
| C seheli | dry | Siluriformes | 0.75 | raw | ina | None | 50.500 | 0.857 | 0.308 | 18.100 | 41.000 | 43.500 | 1.730 |
| C seheli | moist | S salar | 0.86 | raw | ina | None | 50.500 | 0.857 | 0.308 | 18.100 | 41.000 | 43.500 | 1.730 |
| D pastinaca | dry | Fish | 0.77 | raw | ina | H uarnak | 8.650 | 0.732 | 0.248 | 21.200 | 16.800 | 20.200 | 0.679 |
| D pastinaca | moist | Siluriformes | 0.83 | raw | ina | H uarnak | 8.650 | 0.732 | 0.248 | 21.200 | 16.800 | 20.200 | 0.679 |
| E tetradactylum | dry | Siluriformes | 0.75 | raw | ina | None | 39.600 | 0.927 | 0.354 | 18.800 | 65.200 | 19.700 | 1.120 |
| E punctifer | dry | O niloticus | 0.79 | raw | ina | None | 156.000 | 1.190 | 0.338 | 20.400 | 42.800 | 31.600 | 1.500 |
| E punctifer | moist | O niloticus | 0.87 | raw | ina | None | 156.000 | 1.190 | 0.338 | 20.400 | 42.800 | 31.600 | 1.500 |
| E arcuatus | moist | Mytilidae | 0.62 | raw | ufish | Pharella sp. | 154.000 | 8.100 | 0.000 | 8.000 | 0.000 | 36.000 | 2.010 |
| E jordani | dry | Clariidae | 0.76 | raw | ufish | Pleuronectidae | 42.900 | 0.460 | 0.211 | 19.380 | 54.900 | 11.000 | 0.890 |
| E jordani | moist | Siluriformes | 0.84 | raw | ufish | Pleuronectidae | 42.900 | 0.460 | 0.211 | 19.380 | 54.900 | 11.000 | 0.890 |
| E affinis | moist | T trachurus | 0.70 | raw | ina | None | 35.800 | 1.980 | 0.381 | 23.200 | 74.600 | 19.800 | 0.505 |
| E affinis | dry | T trachurus | 0.80 | raw | ina | None | 35.800 | 1.980 | 0.381 | 23.200 | 74.600 | 19.800 | 0.505 |
| H nemurus | dry | Siluriformes | 0.75 | raw | ina | None | 48.800 | 0.596 | 0.385 | 16.300 | 72.400 | 44.500 | 1.400 |
| H nemurus | moist | Siluriformes | 0.83 | raw | ina | None | 48.800 | 0.596 | 0.385 | 16.300 | 72.400 | 44.500 | 1.400 |
| H sagor | dry | Siluriformes | 0.75 | raw | ufish | Siluriformes | 21.000 | 0.300 | 0.410 | 16.300 | 13.000 | 15.000 | 0.510 |
| H sagor | moist | Siluriformes | 0.83 | raw | ufish | Siluriformes | 21.000 | 0.300 | 0.410 | 16.300 | 13.000 | 15.000 | 0.510 |
| I elongata | moist | D labrax | 0.86 | raw | ina | None | 146.000 | 1.660 | 0.845 | 18.500 | 57.300 | 21.200 | 1.280 |
| I elongata | dry | Siluriformes | 0.75 | raw | ina | None | 146.000 | 1.660 | 0.845 | 18.500 | 57.300 | 21.200 | 1.280 |
| I platypterus | dry | Fish | 0.77 | raw | ina | None | 26.400 | 1.080 | 0.259 | 19.900 | 45.800 | 6.600 | 0.458 |
| L crocea | dry | O niloticus | 0.79 | raw | ina | L croceus | 53.000 | 0.700 | 0.000 | 17.700 | 42.570 | 10.000 | 0.580 |
| L crocea | moist | O niloticus | 0.87 | raw | ina | L croceus | 53.000 | 0.700 | 0.000 | 17.700 | 42.570 | 10.000 | 0.580 |
| L vannamei | dry | Not applicable | 1.00 | grilled | ufish | None | 40.000 | 0.900 | 0.190 | 21.200 | 40.000 | 11.000 | 1.530 |
| L vannamei | moist | Not applicable | 1.00 | boiled | ufish | None | 41.000 | 0.900 | 0.200 | 21.900 | 37.000 | 12.000 | 1.580 |
| Loliginidae | moist | Not applicable | 1.00 | boiled | ufish | Loligo spp | 36.000 | 1.000 | 0.000 | 31.200 | 77.000 | 23.000 | 2.470 |
| Loliginidae | dry | Not applicable | 1.00 | grilled | ufish | Loligo spp | 36.000 | 0.900 | 0.000 | 26.100 | 71.000 | 19.000 | 2.470 |
| L bitaeniatus | dry | O niloticus | 0.79 | raw | ina | None | 44.900 | 0.369 | 0.160 | 18.700 | 64.800 | 191.000 | 0.576 |
| L bitaeniatus | moist | O niloticus | 0.87 | raw | ina | None | 44.900 | 0.369 | 0.160 | 18.700 | 64.800 | 191.000 | 0.576 |
| M niger | dry | O niloticus | 0.79 | raw | ina | None | 32.200 | 0.455 | 0.140 | 19.300 | 45.300 | 65.800 | 0.933 |
| M niger | moist | O niloticus | 0.87 | raw | ina | None | 32.200 | 0.455 | 0.140 | 19.300 | 45.300 | 65.800 | 0.933 |
| M rosenbergii | moist | Not applicable | 1.00 | boiled | ufish | None | 25.000 | 0.500 | 0.150 | 20.700 | 30.000 | 10.000 | 1.190 |
| M rosenbergii | dry | Not applicable | 1.00 | grilled | ufish | None | 26.000 | 0.500 | 0.150 | 21.400 | 28.000 | 10.000 | 1.230 |
| M armatus | dry | Siluriformes | 0.75 | raw | ina | None | 312.000 | 1.680 | 0.314 | 16.700 | 29.700 | 12.300 | 1.020 |
| M cordyla | moist | T trachurus | 0.70 | raw | ina | None | 132.000 | 2.160 | 0.408 | 19.400 | 74.700 | 21.000 | 0.655 |
| M cinereus | dry | Siluriformes | 0.75 | raw | ina | None | 53.800 | 1.190 | 0.624 | 18.800 | 57.100 | 9.670 | 0.576 |
| M cinereus | moist | Siluriformes | 0.83 | raw | ina | None | 53.800 | 1.190 | 0.624 | 18.800 | 57.100 | 9.670 | 0.576 |
| M nigriceps | dry | Siluriformes | 0.75 | raw | ina | M alasensis | 786.000 | 3.080 | 0.224 | 15.700 | 62.700 | 97.200 | 2.940 |
| M nigriceps | moist | Siluriformes | 0.83 | raw | ina | M alasensis | 786.000 | 3.080 | 0.224 | 15.700 | 62.700 | 97.200 | 2.940 |
| N thalassina | dry | Siluriformes | 0.75 | raw | ina | None | 36.800 | 0.927 | 0.588 | 17.700 | 45.100 | 11.700 | 0.724 |
| O niloticus | dry | O niloticus | 0.79 | raw | ina | None | 25.900 | 1.790 | 0.407 | 17.300 | 74.200 | 3.770 | 1.910 |
| O marmorata | dry | O niloticus | 0.79 | raw | ina | None | 56.000 | 0.453 | 0.491 | 17.600 | 69.500 | 36.200 | 1.170 |
| O marmorata | moist | Siluriformes | 0.83 | raw | ina | None | 56.000 | 0.453 | 0.491 | 17.600 | 69.500 | 36.200 | 1.170 |
| P hypophthalmus | moist | T trachurus | 0.70 | raw | ina | P pangasius | 14.400 | 0.374 | 0.343 | 15.400 | 109.000 | 9.060 | 1.330 |
| P pangasius | moist | Siluriformes | 0.83 | raw | ina | None | 14.400 | 0.374 | 0.343 | 15.400 | 109.000 | 9.060 | 1.330 |
| P merguiensis | dry | Not applicable | 1.00 | grilled | ufish | P pangasius | 23.000 | 0.100 | 0.000 | 15.300 | 35.000 | 11.000 | 1.090 |
| P merguiensis | moist | Not applicable | 1.00 | boiled | ufish | P pangasius | 24.000 | 0.100 | 0.000 | 15.900 | 33.000 | 12.000 | 1.090 |
| P argentata | moist | Siluriformes | 0.83 | raw | ina | P aneus | 199.000 | 0.904 | 0.271 | 19.300 | 71.500 | 20.000 | 1.090 |
| P argentata | dry | S salar | 0.86 | raw | ina | P aneus | 199.000 | 0.904 | 0.271 | 19.300 | 71.500 | 20.000 | 1.090 |
| Plotosidae | dry | Siluriformes | 0.75 | raw | ufish | P hypophthalmus | 17.000 | 0.100 | 0.080 | 17.800 | 15.000 | 12.000 | 0.330 |
| Plotosidae | moist | Not applicable | 1.00 | boiled | ufish | P hypophthalmus | 14.000 | 0.100 | 0.070 | 14.700 | 13.000 | 11.000 | 0.270 |
| P erosa | dry | Mollusca | 0.61 | raw | ufish | Verenidae | 290.000 | 6.600 | 0.000 | 9.200 | 110.000 | 79.000 | 1.170 |
| P erosa | moist | Not applicable | 1.00 | boiled | ufish | Verenidae | 290.000 | 8.400 | 0.000 | 14.600 | 160.000 | 113.000 | 1.850 |
| P maculatus | dry | O niloticus | 0.79 | raw | ina | None | 44.800 | 0.605 | 0.275 | 18.400 | 36.800 | 108.000 | 1.260 |
| Rastrelliger | moist | Atlantic horse mackerel | 0.70 | raw | ina | R kanagurta | 204.000 | 2.970 | 0.376 | 20.800 | 47.000 | 19.200 | 1.190 |
| Rastrelliger | dry | Atlantic horse mackerel | 0.80 | raw | ina | R kanagurta | 204.000 | 2.970 | 0.376 | 20.800 | 47.000 | 19.200 | 1.190 |
| R trichopterus | dry | S. solea | 0.72 | raw | ina | T trichopterus | 117.000 | 1.440 | 0.275 | 18.200 | 40.900 | 25.300 | 2.880 |
| S pilchardus | dry | Atlantic horse mackerel | 0.80 | raw | ufish | Sardina sp. | 198.000 | 1.550 | 1.320 | 19.800 | 51.000 | 10.000 | 0.700 |
| S pilchardus | moist | Not applicable | 1.00 | preserved | ufish | Sardina sp. | 455.000 | 2.690 | 2.980 | 18.500 | 39.000 | 56.000 | 2.000 |
| S argus | moist | D labrax | 0.86 | raw | ina | None | 82.500 | 1.030 | 0.243 | 18.100 | 36.900 | 23.400 | 1.510 |
| S argus | dry | Siluriformes | 0.75 | raw | ina | None | 82.500 | 1.030 | 0.243 | 18.100 | 36.900 | 23.400 | 1.510 |
| S commerson | moist | T trachurus | 0.70 | raw | ina | None | 26.300 | 0.651 | 0.292 | 21.000 | 99.100 | 15.400 | 0.561 |
| S commerson | dry | T trachurus | 0.80 | raw | ina | None | 26.300 | 0.651 | 0.292 | 21.000 | 99.100 | 15.400 | 0.561 |
| S serrata | moist | Not applicable | 1.00 | boiled | ufish | None | 178.000 | 1.200 | 0.000 | 20.000 | 67.000 | 61.000 | 3.500 |
| S serrata | dry | Not applicable | 1.00 | grilled | ufish | None | 176.000 | 1.100 | 0.000 | 19.700 | 59.000 | 60.000 | 3.500 |
| Sepiida | moist | Not applicable | 1.00 | boiled | ufish | None | 31.300 | 4.000 | 0.720 | 31.300 | 87.000 | 39.000 | 5.850 |
| Sepiida | dry | Not applicable | 1.00 | grilled | ufish | None | 26.200 | 3.500 | 0.580 | 26.200 | 81.000 | 32.000 | 4.890 |
| S taty | dry | O niloticus | 0.79 | raw | ina | None | 290.000 | 1.820 | 1.110 | 19.000 | 37.100 | 6.980 | 2.120 |
| S taty | moist | O niloticus | 0.87 | raw | ina | None | 290.000 | 1.820 | 1.110 | 19.000 | 37.100 | 6.980 | 2.120 |
| Sphyraena | dry | Northern pike | 0.86 | raw | ina | S jello | 15.300 | 0.310 | 0.187 | 19.300 | 39.800 | 40.200 | 0.586 |
| Sphyraena | moist | Siluriformes | 0.83 | raw | ina | S jello | 15.300 | 0.310 | 0.187 | 19.300 | 39.800 | 40.200 | 0.586 |
| S gracilis | moist | T trachurus | 0.70 | raw | ina | None | 571.000 | 4.090 | 0.423 | 20.200 | 86.000 | 10.200 | 2.240 |
| S gracilis | dry | T trachurus | 0.80 | raw | ina | None | 571.000 | 4.090 | 0.423 | 20.200 | 86.000 | 10.200 | 2.240 |
| T anomala | dry | Not applicable | 1.00 | grilled | ufish | True lobster | 77.000 | 1.200 | 0.190 | 21.100 | 73.000 | 7.000 | 2.620 |
| Toxotes | dry | O niloticus | 0.79 | raw | ina | None | 135.000 | 1.480 | 0.578 | 17.400 | 38.000 | 36.900 | 1.230 |
| T microlepis | dry | O niloticus | 0.79 | raw | ina | T chatareus | 135.000 | 1.480 | 0.578 | 17.400 | 38.000 | 36.900 | 1.230 |
| T microlepis | moist | O niloticus | 0.87 | raw | ina | T chatareus | 135.000 | 1.480 | 0.578 | 17.400 | 38.000 | 36.900 | 1.230 |
| T lepturus | moist | T trachurus | 0.70 | raw | ina | S commerson | 26.300 | 0.651 | 0.292 | 21.000 | 99.100 | 15.400 | 0.561 |
| T lepturus | dry | T trachurus | 0.80 | raw | ina | S commerson | 26.300 | 0.651 | 0.292 | 21.000 | 99.100 | 15.400 | 0.561 |
| V cochlidium | moist | Mytilidae | 0.62 | raw | ufish | T articulatus | 91.000 | 3.200 | 0.000 | 22.700 | 79.200 | 0.000 | 2.890 |
| V cochlidium | dry | Mollusca | 0.61 | raw | ufish | T articulatus | 91.000 | 3.200 | 0.000 | 22.700 | 79.200 | 0.000 | 2.890 |
| W attu | moist | Siluriformes | 0.83 | raw | ina | None | 99.400 | 0.623 | 0.280 | 15.100 | 134.000 | 5.190 | 0.359 |

uFiSh, FAO/INFOODS Databases, Fish Base Nutrient Indonesian dataset.

Not applicable, the cooking methods are already accounted in the nutrient value.

**Supplementary Information Table S3**. Conversion factors to calculate wet weight equivalent from edible weight

| Species | Factor |  | Species | Factor |
| --- | --- | --- | --- | --- |
| Acetes | 0.9 |  | N. thalassina | 0.89 |
| A. chacunda | 0.9 |  | O. niloticus | 0.8 |
| A. japonicus | 0.8 |  | O. marmorata | 0.8 |
| C. lucius | 0.8 |  | P. hypophthalmus | 0.8 |
| C. micropeltes | 0.8 |  | P. pangasius | 0.8 |
| C. chanos | 0.8 |  | P. merguiensis | 0.68 |
| C. seheli | 0.8 |  | P. argentata | 0.8 |
| D. pastinaca | 0.8 |  | Plotosidae | 0.8 |
| E. tetradactylum | 0.8 |  | P. erosa | 0.2 |
| E. punctifer | 1 |  | P. maculatus | 0.8 |
| E. arcuatus | 0.2 |  | Rastrelliger | 0.8 |
| E. jordani | 0.8 |  | R. trichopterus | 0.8 |
| E. affinis | 1 |  | S. pilchardus | 1 |
| H. nemurus | 0.8 |  | S. argus | 0.8 |
| H. sagor | 0.8 |  | S. commerson | 0.8 |
| I. elongata | 0.8 |  | S. serrata | 0.45 |
| I.platypterus | 0.8 |  | Sepiida | 1 |
| L. crocea | 0.8 |  | S. taty | 0.8 |
| L. vannamei | 0.68 |  | Sphyraena | 0.8 |
| Loliginidae | 1 |  | S. gracilis | 0.8 |
| L. bitaeniatus | 0.8 |  | T. anomala | 0.68 |
| M. niger | 0.8 |  | Toxotes | 0.8 |
| M. rosenbergii | 0.68 |  | T. microlepis | 0.8 |
| M. armatus | 0.8 |  | T. lepturus | 0.8 |
| M. cordyla | 0.8 |  | V. cochlidium | 0.2 |
| M. cinereus | 0.8 |  | W. attu | 0.8 |
| M. nigriceps | 0.8 |  |  |  |

Wet weight equivalent = edible weight/ factor
